# Cognitive Impairment Impacts Exercise Effects on Cognition in Multiple Sclerosis

**DOI:** 10.3389/fneur.2020.619500

**Published:** 2021-01-28

**Authors:** Annette Rademacher, Niklas Joisten, Sebastian Proschinger, Wilhelm Bloch, Roman Gonzenbach, Jan Kool, Dawn Langdon, Jens Bansi, Philipp Zimmer

**Affiliations:** ^1^Department for Molecular and Cellular Sports Medicine, Institute of Cardiovascular Research and Sports Medicine, German Sport University, Cologne, Germany; ^2^Department of “Performance and Health (Sports Medicine)”, Institute of Sport and Sport Science, Technical University Dortmund, Dortmund, Germany; ^3^Department of Neurology, Clinics of Valens, Rehabilitation Centre Valens, Valens, Switzerland; ^4^Department of Psychology Health and Well-Being Clinical, Health and Social Psychology Royal Holloway University London, London, United Kingdom

**Keywords:** cognitive performance, exercise, processing speed, verbal learning, visuospatial memory, high-intensity exercise

## Abstract

**Purpose:** Exercise training reveals high potential to beneficially impact cognitive performance in persons with multiple sclerosis (pwMS). Research indicates that high-intensity interval training (HIIT) has potentially higher effects on physical fitness and cognition compared to moderate continuous exercise. This study (i) compares the effects of a 3-week HIIT and moderate continuous exercise training on cognitive performance and cardiorespiratory fitness of pwMS in an overall analysis and (ii) investigates potential effects based on baseline cognitive status in a subgroup analysis.

**Methods:** Seventy-five pwMS were randomly assigned to an intervention (HIIT: 5 × 1.5-min intervals at 95–100% HR_max_, 3 ×/week) or active control group (CG: 24 min continuous exercise at 65% HR_max_, 3 ×/week). Cognitive performance was assessed pre- and post-intervention with the Brief International Cognitive Assessment for MS (BICAMS). (I) To examine potential within (time) and interaction (time × group) effects in the overall analysis, separate analyses of covariance (ANCOVA) were conducted. (II) For the subgroup analysis, participants were divided into two groups [intact cognition or impaired cognition (>1.5 standard deviation (SD) compared to healthy, age-matched norm data in at least one of the three tests of the BICAMS]. Potential impacts of cognitive status and intervention were investigated with multivariate analyses of variance (MANOVA).

**Results:** Overall analysis revealed significant time effects for processing speed, verbal learning, rel. VO2peak, and rel. power output. A time^*^group interaction effect was observed for rel. power output. Subgroup analysis indicated a significant main effect for cognition (impaired cognition vs. intact cognition). Subsequent *post-hoc* analysis showed significant larger effects on verbal learning in pwMS with impaired cognition.

**Conclusion:** Current results need to be confirmed in a powered randomized controlled trial with cognitive performance as primary endpoint and eligibility based on cognitive performance that is assessed prior to study inclusion.

## Introduction

Cognitive impairment represents a common and debilitating symptom in multiple sclerosis (MS). Forty-three percent to 70% of persons with MS (pwMS) experience cognitive impairment, predominantly characterized by slowed processing speed and impaired memory function (1). Since reduced physical ability is often described as a hallmark of MS symptomology, cognitive impairment tends to lose focus in everyday care. Nevertheless, impaired cognition has a profound impact on peoples' working and driving ability and on their overall quality of life (2).

Existing pharmacological treatments target a reduction of disease activity by modifying the immune system and its effects on the central nervous system (CNS). A few of these disease-modifying drugs reveal cognition-enhancing effects (3). However, they are not generally effective in counteracting cognitive impairment (4). Moreover, symptomatic treatments that are used for dementia are not, or only marginally effective for cognitive impairment in pwMS (5). Against this backdrop, investigations on novel non-pharmacological treatment options gain focus in current research.

Exercise training especially became of particular interest as a non-pharmacological supportive treatment option in the last decade. Previous research has already shown associations between exercise training and improved cognitive performance in healthy and cognitively impaired older adults (4, 6, 7). Additionally, data also suggest exercise-induced neuroprotective effects in several neurological diseases, such as Alzheimer's disease (8).

In contrast, little is known about the effects of exercise training on degenerative CNS processes in MS and its impact on cognitive impairment. Currently, research on this topic is growing and several approaches investigating potential beneficial effects of exercise for pwMS have been initiated. Research indicates positive associations between an increased cardiovascular fitness (VO2peak) and larger volumes of deep gray matter structures, involving the hippocampus (9). The hippocampus is indeed mainly responsible for memory and learning, functions that are commonly affected in MS. Another study revealed increased cortical thickness following an exercise training intervention, indicating neuroprotective and potential neuroregenerative effects of exercise (10). In fact, high-intensity interval training (HIIT) has been described to potentially induce greater enhancements in cardiorespiratory fitness than moderate continuous exercise in pwMS (11). Moreover, Zimmer et al. (12) showed in a previous randomized controlled trial (RCT) that HIIT significantly improved verbal learning compared to a moderate continuous control group (CG).

On a functional level, a growing body of literature has investigated the effects of exercise training on cognitive performance in pwMS. However, existing results remain contradictory, since some studies report beneficial impacts on specific cognitive domains such as verbal learning (12) while others demonstrate non-significant results (13). Overall, evidence of exercise studies on cognitive performance in pwMS is still sparse. A recent meta-analysis evaluating the effects of exercise training on global cognitive performance and MS-specific cognitive domains (processing speed, learning/memory, executive functions, and attention) (14) did not identify any significant effects. This work supports the conclusions of a former meta-analysis and review (15, 16) with regard to several, still emerging, methodological limitations of existing studies. In addition to many other limitations, most of the existing studies investigating exercise-induced effects on cognitive performance do not focus on screening participants' cognitive performance prior to inclusion.

The objective of this study is to analyze the effects of a HIIT and moderate continuous exercise on cognitive performance in pwMS. Since cognitive performance was a secondary outcome of this RCT (17), the above mentioned limitation of participants not being included based on their cognitive impairment is given. In order to go one step further and consider this limitation, we not only investigate (i) the effect of HIIT on cognitive performance of the total sample (overall analysis) but additionally (ii) conduct a subgroup analysis (total sample subdivided based on baseline cognitive status) in order to achieve more meaningful results on this secondary outcome.

## Methods

### Study Design and Overview

The original study is a RCT with a parallel (1:1) group design and primarily investigated the change of proportions of circulating T-regulatory cells (Tregs) over a 3-week intervention period comparing HIIT vs. CG. The study was approved by the regional ethics committee (EKOS18/96; Project ID: 2018–01378), registered at ClinicalTrials.gov (NCT03652519; August 29, 2018) prior to recruitment start and conducted in accordance with the principles of the Declaration of Helsinki. Details on methods and all outcomes that are not relevant for the present investigation are shown elsewhere (17). This publication presents an analysis of this RCT with special interest on the secondary outcome cognitive performance.

### Participant Recruitment and Eligibility

Participant recruitment, testing, and exercise intervention were conducted in the inpatient rehabilitation clinic Valens (Switzerland). Inpatients were screened for eligibility over a 12-month period (October 2018–October 2019). All inpatients received a comprehensive medical check on the day of admission. Persons >21 years old holding a definite MS diagnosis [according to the revised McDonald criteria (18)] with a relapsing–remitting or secondary progressive disease course and an Expanded Disability Status Scale (EDSS) score between 3.0 and 6.0 (inclusive) fulfilled the key inclusion criteria. Persons with concomitant diseases (internistic, orthopedic, neurological, acute melanoma, and cancer), acute relapses, or disease worsening immediately before study start, limiting the participation in the exercise intervention or affecting study outcomes, were excluded. Moreover, non-German-speaking persons and persons with diagnosed psychological disorders were excluded, since the understanding of study course and execution of instructions could be affected. Pregnancy or breast feeding, drug or alcohol abuse, and persons employed for study execution were also criteria for study exclusion (17). Additionally, participants who experienced acute relapses or received immune-modulatory medication the day prior to cardiopulmonary exercise testing (CPET) were excluded. In case participants developed acute unwellness over the study period, exercise sessions were canceled on that day and if possible conducted on another day of the week. Participants were informed about the study and gave their written consent before inclusion.

### Randomization and Masking

After baseline assessment, participants were randomized (1:1) into an exercise intervention group or CG. A concealed randomization was conducted with the “Randomization-In-Treatment-Arms” software (RITA, Evident, Germany). Cardiorespiratory fitness (assessed by CPET), disease severity (EDSS score), age, and fatigue [Fatigue Scale for Motor and Cognitive Functions (FSMC) (19)] were applied as factors for stratification. For all stratification factors, separate ranges were defined in the randomization software prior to first randomization (EDSS: 3/3.5, 4/4.5, 5/5.5, 6; cardiorespiratory fitness: <100 W, ≥100 W, age: 20–29, 30–39, 40–49, 50–59, 60–69, 70–80, fatigue: <43, ≥43). Randomization was carried out by a researcher at the German Sport University Cologne, who was not involved in the study procedures during data recruitment. CPET was conducted by the principal investigator who was blinded to the training condition.

### Exercise and Control Group Treatment

The exercise interventions consisted of aerobic endurance training sessions on a bicycle ergometer. Both groups exercised three times a week for 3 weeks. Exercise intensity was heart rate controlled based on the highest heart rate (HR_max_) achieved at baseline CPET. Each session comprised a 3-min warm-up and cool-down period at low intensity [50% maximum heart rate (HR_max_)]. Besides the exercise intervention, participants of both groups received the regular individual rehabilitation program of the Valens clinic.

#### Experimental Intervention Group (HIIT)

The exercise group performed five 1.5-min high-intensity intervals at 95–100% of HR_max_ with 80–100 rpm. Between the intervals, active breaks of 2 min unloaded pedaling were conducted, aiming to achieve 60% HR_max_.

#### Control Group Treatment

Participants assigned to the CG exercised continuously three times a week for 24 min at 65% of HR_max_ with 60–70 rpm. This intervention represents the usual exercise regime of the Valens clinic and can be described as a standard care active control regime.

### Outcome Measures

Outcome measures were assessed after the day of clinical admission, prior to intervention start (T0) and at discharge of the 3-week intervention (T1).

#### Aerobic Fitness

Participants performed a graded cardiopulmonary exercise (Jaeger CPX, Germany) test at T0 and T1 on a bicycle ergometer (Ergoline 800, Germany) until a participants' symptom reached maximum (e.g., muscular fatigue). Peak oxygen consumption (VO2peak), maximum workload (watts), and heart rate [beats per minute (bpm)] were assessed during the test. The protocol started with 3 min of rest (no pedaling), 3 min of unloaded pedaling (warm-up), followed by the testing, and ended with 3 min of unloaded pedaling (cool-down). Workload was continuously ramp-type increased by 10 W each minute to ensure a testing phase of 8–12 min. Baseline CPET results (HR_max_) served as the anchor for individual exercise intensities in the HIIT group and CG.

#### Patient-Reported Outcome Measures

Fatigue was measured with the German version of the FSMC (19) comprising 10 items for motor and 10 items for cognitive fatigue. Cutoff scores for low and high levels of fatigue were set at 43/100 for the total score, 22/50 for the motor (FSMC mot.), and 22/50 for the cognitive (FSMC cog.) subscores.

#### Cognitive Performance

Cognitive performance was assessed with the Brief International Cognitive Assessment for MS (BICAMS) (1) modified for the use in German language. This test battery contains three tests assessing the main cognitive domains vulnerable to MS. Processing speed is measured by the Symbol Digit Modalities Test (SDMT), verbal learning by the Verbal Learning Memory Test (VLMT), and visuospatial learning and memory by the Brief Visuospatial Memory Test-Revised (BVMT-R). The original BICAMS version recommended the California Verbal Learning Test or any verbal memory list learning task. The VLMT was used in this study, because the VLMT norm data for the German population are based on a larger sample size and include a larger age range (20). Parallel versions for two tests, the VLMT, and the BVMT-R, were applied. The BICAMS test battery represents a validated, frequently recommended and applied test battery to evaluate cognitive performance of the most commonly affected domains in pwMS. Therefore, only this assessment was used for the current analysis.

### Statistical Analysis

Sample size calculation focused on detecting between group effects on the proportion of Tregs, the primary outcome of the RCT. Details on the precise process of sample size calculation are explained elsewhere (17). The final sample size for this study results in *N* = 72 participants.

In a first step, an overall analysis was conducted with separate analysis of covariance models with repeated measures and adjusted for baseline values (ANCOVA) to assess potential between-group effects (HIIT vs. CG) over time for cognitive performance, fatigue, and cardiorespiratory fitness outcomes. Therefore, “time” was defined as the within-subject factor and “group” was defined as the between-subject factor. Dependent variables were the cognitive outcomes (SDMT, VLMT, and BVMT-R), the fatigue outcome (FSMC), and the cardiorespiratory fitness outcomes [rel. (relative) VO2peak and rel. power output]. In this analysis, the whole sample was analyzed as one.

In a second step, MAN(C)OVA was conducted to determine potential effects of cognitive status (impaired cognition vs. intact cognition) and group (HIIT vs. CG) and their interaction (group*cognition) on changes of cognitive performance. For this subgroup analysis, the sample was divided into two groups, “impaired cognition” and “intact cognition.” Participants with baseline values >1.5 standard deviation (SD) compared to healthy, age-matched norm data (21–23) in at least one of the three tests were allocated to the “impaired cognition” group. All other participants were allocated to the “intact cognition” group. For the multivariate ANOVAs, the delta values of the SDMT, VLMT, and BVMT-R were used as the dependent variable and the factors “group” and “cognition” (impaired cognition/intact cognition) were used as fixed factors. Box's Test of Equality of Covariance Matrices and Levene's Test were checked throughout the analysis. An additional MANOVA was conducted adjusted for levels of fatigue since it might be a confounding factor.

Potential baseline differences were assessed with independent *t-*tests and Fisher's exact test and univariate one-way ANOVAs. All analyses were conducted with the intention-to-treat analysis (ITT); therefore, all randomized participants were included in the analysis. Missing values were imputed with the last observation carried forward method (LOCF), using baseline values. Outliers defined as *z* scores </>3 were replaced by the cutoff value of 3 SD (mean ± 3 × SD) from the mean score of the concerned variable. Significance was defined as *p* ≤ 0.05 for univariate ANOVAs and main effects of MANOVAs. Correcting for multiple testing, the significance level for the subsequent ANOVA analysis of the MANOVAs was reduced to *p* ≤ 0.017. All outcome measures of the ANCOVAs and the MANOVA are presented with *p-*values, *F* (df), and effect sizes (partial η^2^). All statistical procedures were conducted with SPSS 26® (IBM®, Armonk, NY, USA).

## Results

**Figure 1:**
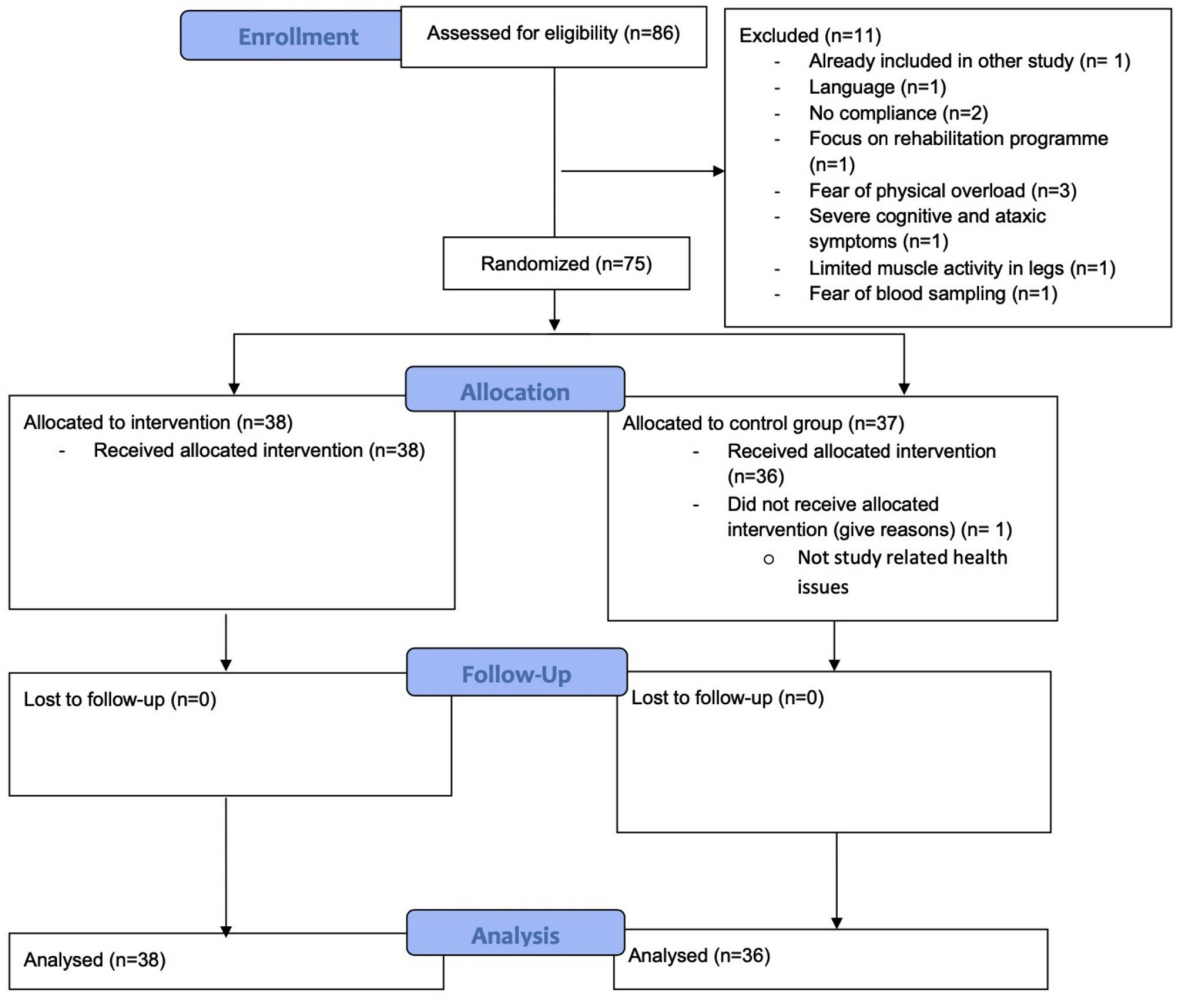
Participant flow diagram.

A total of 75 participants were included in the study and 74 participants completed this study, leading to a completion rate of 98.67%. All participants exercised and were analyzed according to their randomized group. One participant of the CG dropped out due to non-study-related health issues following a surgery prior to baseline CPET. The overview of the study flow is shown in Figure 1.

No adverse events occurred. One participant declined the cognitive assessments, so the total number of participants in the subgroup analysis for cognitive performance was reduced to 73. From the 74 participants that completed the study, data of cognitive performance (all three tests) and fatigue (FSMC cog. and FSMC total) were imputed each for one participant. The reason for this missing cognitive data was that one participant declined to take part in the cognitive assessments at t1 because they felt uncomfortable. Data of the FSMC were imputed, because one question was declined by the participant. Data of both subscales of the HADS are missing for one participant and data of the anxiety subscale are missing for another participant, because items were not answered. Baseline and clinical characteristics of the participants are shown in Table 1. Except for sex (in the subgroup analysis), no baseline differences between groups were found, neither within the overall nor the subgroup analysis (Table 1). In total, 70.6% of the total sample of the impaired participants were classified as impaired in only one test of the BICAMS test battery (50% in SDMT, 50% in BVMT-R). The remaining 29.4% were classified as impaired in two or more tests (14.7% in two tests, 14.7% in three tests). Eighty percent of those who were classified as impaired in two tests showed deficits in the SDMT and BVMT-R test and 20% showed deficits in the SDMT and VLMT test. With regard to the attendance rates, participants of the HIIT group reached, on average, 79%, and those in the CG reached 70% of the planned exercise sessions. Adherence rates in the subgroups were 77% for HIIT + impaired cognition, 81% for HIIT + intact cognition, 72% for CG + impaired cognition, and 67% for CG + intact cognition. This analysis was conducted based on the intention-to-treat method, consequently including all training sessions independent of the number of missed sessions. No differences between attendance rates of the groups within the overall or subgroup analysis exist (overall analysis: 0.067; subgroup analysis: 0.268). The average training intensity of the HIIT group was 98% HR_max_ and that of the CG was 77% HR_max_. In the subgroups: HIIT + impaired cognition, 97% HR_max_; HIIT + intact cognition, 99% HR_max_; CG + impaired cognition, 80% HR_max_; CG + intact cognition, 75% HR_max_. Ninety-two percent of the exercise sessions in the HIIT group fulfilled the targeted interval time. For the CG, on average, 94% of the planned exercise was fulfilled.

**Table 1:** Baseline characteristics of the participants.

	**Overall analysis**			**Subgroup analysis**				
				**Participants with impaired cognition**		**Participants with intact cognition**		
	**HIIT (*n* = 38)**	**CG (*n* = 36)**	***p***	**HIIT (*n* = 15)**	**CG (*n* = 19)**	**HIIT (*n* = 23)**	**CG (*n* = 16)**	***p***
Sex (f/m)	27/11	21/15	0.331	15/0	9/10	12/11	12/4	0.002^*^
Age (years)	51 (10.97)	49 (10.12)	0.418	50.73 (13.52)	49.26 (10.44)	51.17 (9.27)	48.31 (10.25)	0.843
MS phenotype (RRMS/SPMS)	23/15	22/14	1.000	9/6	12/7	14/9	9/7	0.987
EDSS-Score	4.5 (1.05)	4.53 (1.08)	0.911	4.67 (1.11)	4.5 (1.2)	4.39 (1.01)	4.59 (0.99)	0.876
Rel. VO2peak (ml kg^−1^ min^−1^)	19.04 (5.61)	19.25 (5.12)	0.868	16.79 (4.68)	19.38 (5.8)	20.51 (5.77)	19.04 (4.54)	0.223
Rel. power output (watts/kg)	1.34 (0.53)	1.32 (0.44)	0.866	1.22 (0.42)	1.36 (0.47)	1.43 (0.58)	1.26 (0.42)	0.559
Power output (watts)	96.59 (38.87)	95.50 (31.42)	0.894	81.33 (31.78)	97.11 (31.01)	106.55 (40.46)	90.5 (30.94)	0.163
Fatigue (FSMC)	69.45 (15.66)	66.42 (13.41)	0.373	71.28 (17.64)	70.05 (12.78)	68.26 (14.52)	63.5 (12.82)	0.448
Motor fatigue (FSMC-mot)	36.73(7.92)	35.75 (6.85)	0.571	35.85 (8.91)	36.74 (6.94)	37.3 (7.36)	35.44 (6.13)	0.863
Cognitive fatigue (FSMC-cog)	32.26 (9.68)	30.67 (7.84)	0.440	34.27 (9.88)	33.32 (6.95)	30.96 (9.54)	28.06 (8.02)	0.187
HADS (Depression subscale)	4.66 (3.31)	4.00 (3.26)	0.402	4.73 (3.37)	4.90 (4.08)	4.61 (3.35)	3.06 (1.81)	0.356
HADS (Anxiety subscale)	5.47 (3.61)	4.62 (3.62)	0.318	6.33 (3.37)	5.47 (3.94)	4.91 (3.73)	4.00 (3.10)	0.326
SDMT (points)	43.44 (9.95)	42.63 (13.76)	0.771	37.8 (8.41)	32.84 (7.23)	47.13 (9.25)	54.25 (10.04)	
VLMT (points)	52.08 (8.95)	52.37 (11.73)	0.906	48.6 (8.67)	47.37 (10.48)	54.35 (8.56)	58.31 (10.51)	
BVMT-R (points)	20.42 (7.4)	20.09 (7.95)	0.853	14.93 (5.26)	15.89 (6.91)	24 (6.37)	25.06 (6.09)	

*Data are presented as mean (standard deviation) except for sex and MS phenotype (proportions). HIIT, high-intensity group; CG, control group; MS, multiple sclerosis; RRMS, relapsing-remitting multiple sclerosis; SPMS, secondary progressive multiple sclerosis; EDSS, Expanded Disability Status Scale; rel., relative; FSMC, Fatigue Scale for Motor and Cognitive Functions; mot., motor subscale; cog., cognition subscale; HADS, Hospital Anxiety and Depression Scale*.

* *Significant differences between groups*.

Analysis of cardiorespiratory fitness (HIIT vs. CG) showed significant effects for the main factor time (time effects) for rel. VO2peak and rel. power output. Significant interaction effects (time × group) were only observed for the rel. power output. Bonferroni-corrected *post-hoc* tests showed an improvement over time for both groups in levels of rel. VO2peak (HIIT: *p* < 0.001; 95% CI [1.697; 3.371]; CG: *p* < 0.001; 95% CI [0.741; 2.461]). Moreover, *post-hoc* tests showed that the HIIT group had significant higher rel. power outputs compared to the CG at t1. (*p* = 0.011; 95% CI [0.034; 0.250]). For the outcome fatigue, no time (*p* = 0.305) or interaction effects (*p* = 0.404) could be observed. ANCOVA results are listed in Table 2.

**Table 2:** ANCOVA results of the overall analysis (HIIT vs. CG).

	**Descriptive analysis**				**ANCOVA**	
	**HIIT (*****n*** **=** **38)**		**CG (*****n*** **=** **36)**		**Time *p*-Value** ***F*-Value (df = 1)** **Partial η^**2**^**	Group^*^Time *p-*Value ***F-*Value (df = 1)** **Partial η^**2**^**
	**T0**	**T1**	**T0**	**T1**		
SDMT (points)	43.44	47.11	42.63	46.43	0.040^*^	0.912
	(9.95)	(10.36)	(13.76)	(14.76)	4.367	0.012
					0.059	0.000
VLMT (points)	52.08	52.74	52.37	52.77	0.003^*^	0.914
(Total score trials 1–5)	(8.95)	(10.87)	(11.73)	(10.3)	9.522	0.012
					0.120	0.000
BVMT-R (points)	20.42	19.89	20.09	19.26	0.000^*^	0.718
	(7.4)	(6.55)	(7.95)	(7.25)	17.827	0.131
					0.203	0.002
Rel. VO2peak (mL kg^−1^ min^−1^)	19.04	21.58	19.25	20.84	0.001^*^	0.126
	(5.61)	(5.84)	(5.12)	(5.2)	11.101	2.401
					0.135	0.033
Rel. power output (watts/kg)	1.34	1.63	1.32	1.47	0.000^*^	0.011^*^
	(0.53)	(0.54)	(0.44)	(0.45)	19.077	6.869
					0.212	0.088
Fatigue (FSMC)	69.45	66.42	62.84	61.67	0.305	0.404
	(15.66)	(13.41)	(17.03)	(17.60)	1.066	0.704
					0.015	0.010

*Data are presented as mean (standard deviation). HIIT, high-intensity group; CG, control group; T0, baseline; T1, post-intervention; SDMT, Symbol Digit Modalities Test; VLMT, Verbal Learning Memory Test; BVMT-R, Brief Visuospatial Memory Test-Revised; rel., relative; FSMC, Fatigue Scale for Motor and Cognitive Functions*.

* Significant main effect (time) or interaction (time*group).

Regarding outcomes of cognitive performance, two separate analyses were conducted. (I) The overall (HIIT vs. CG) analysis revealed significant time effects for processing speed (SDMT), verbal learning (VLMT), and visuospatial memory (BVMT-R) but no significant group or group × time interaction. ANCOVA results are listed in Table 2.

**Figure 2:**
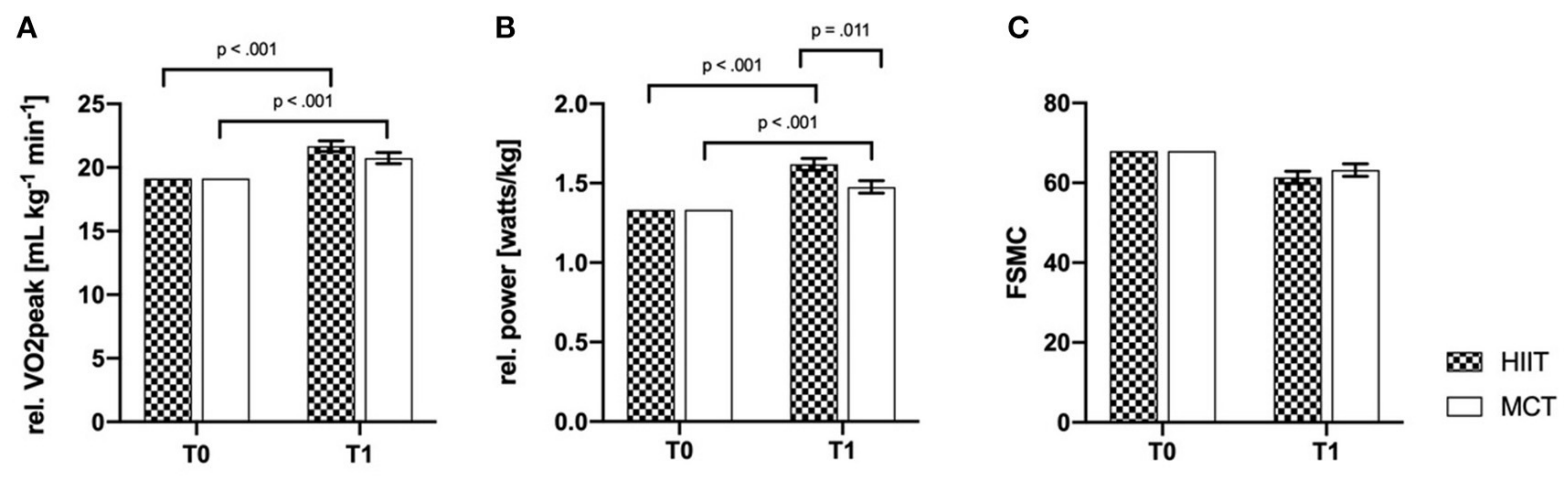
Baseline-adjusted ANCOVA results for physical fitness outcomes **(A, B)** and fatigue **(C)** for the intervention (HIIT) and control group (CG). T0, baseline; T1, post-intervention; FSMC, fatigue scale for motor and cognitive functions; rel., relative. Deviation bars are shown as standard error.

**Figure 3:**
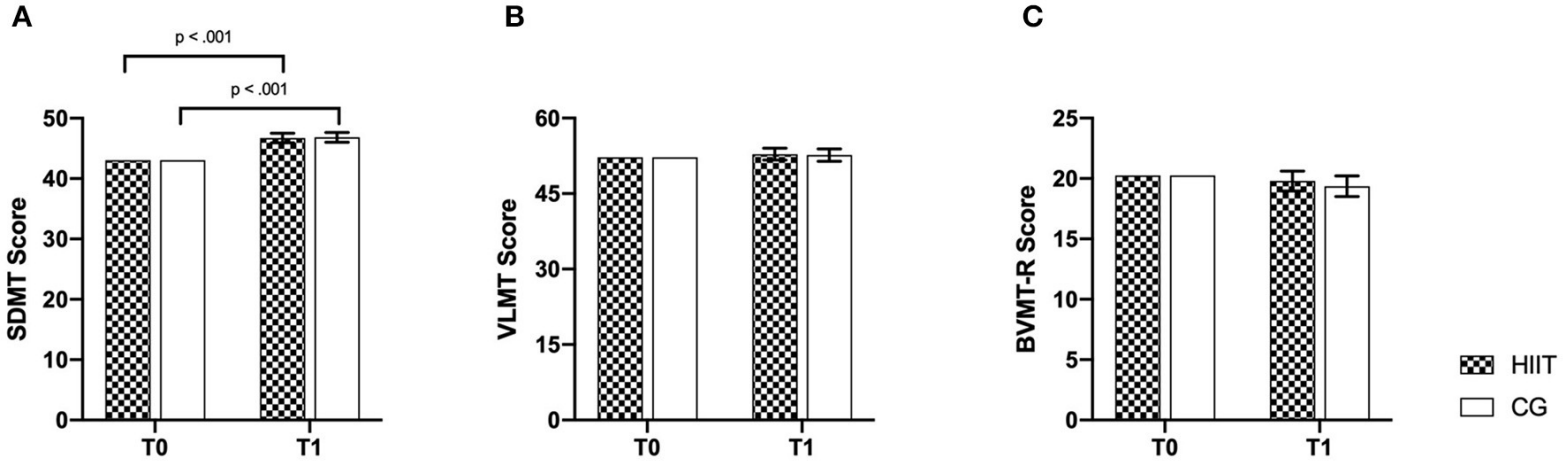
Baseline-adjusted ANCOVA results for cognitive performance parameters for the intervention (HIIT) and control group (CG). T0, baseline; T1, post-intervention. **(A)** SDMT, Symbol Digit Modalities Test; **(B)** VLMT, Verbal Learning Memory Test; **(C)** BVMT-R, Brief Visuospatial Memory Test-Revised. Deviation bars are shown as standard error.

**Figure 4:**
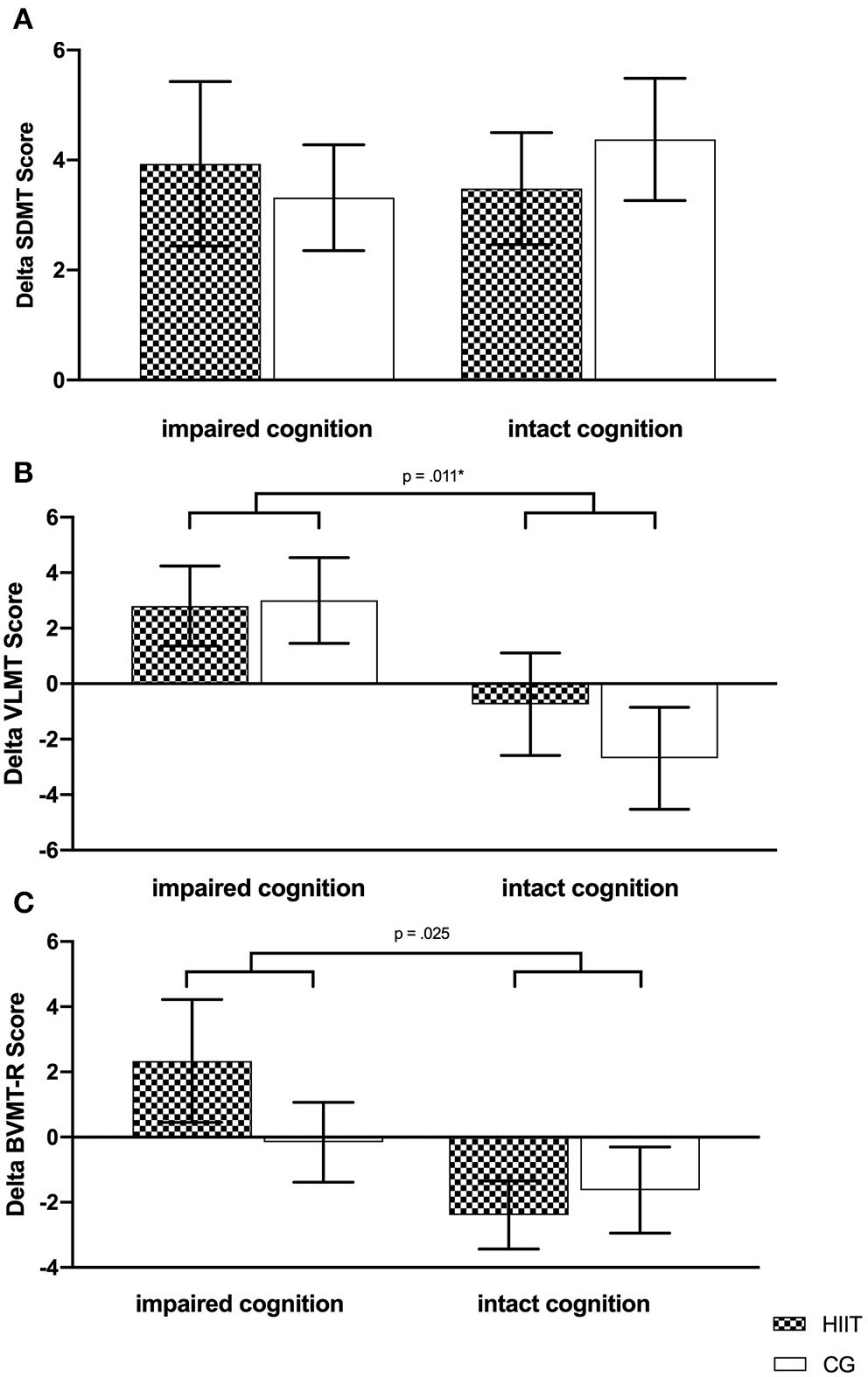
Changes of cognitive performance parameters based on baseline cognition for the intervention (HIIT) and control group (CG). **(A)** SDMT, Symbol Digit Modalities Test; **(B)** VLMT, Verbal Learning Memory Test; **(C)** BVMT-R, Brief Visuospatial Memory Test-Revised. Deviation bars are shown as standard error. *Significant changes between groups of cognitive status.

Bonferroni-corrected *post-hoc* tests showed improvements of processing speed (HIIT: *p* < 0.001; 95% CI [2.112; 5.223]; CG: *p* < 0.001; 95% CI [2.172; 5.414]) over time in both groups; VLMT and BVMT-R showed no effects. For the variables VLMT and BVMT-R, no significant results were observed after Bonferroni-corrected *post-hoc* tests {VMLT (HIIT: *p* = 0.60; 95% CI [−1.731; 2.977]; CG: *p* = 0.723; 95% CI [−2.015; 2.891]), BVMT-R (HIIT: *p* = 0.577; 95% CI [−2.114; 1.186]; CG: *p* = 0.302; 95% CI [−2.616; 0.823])}. Baseline-adjusted ANCOVA results for all outcomes of the overall analysis are shown in Figures 2, 3.

(II) MANOVA results of the subgroup analysis revealed a significant main effect for cognition (impaired cognition vs. intact cognition) but not for the main factor group or their interaction (cognition × group). Subsequent *post-hoc* analysis revealed significant differences between impaired cognition and intact cognition for verbal learning (impaired cognition: 95% CI [0.345; 5.455] intact cognition 95% CI [−4.121; 0.695]). Since the level of significance was corrected for multiple testing, *p-*value was reduced to 0.017. Therefore, no further significant effects were detected. However, a tendency (*p* = 0.025) could be observed for the visuospatial memory (impaired cognition: 95% CI [−0.871; 3.047]; intact cognition 95% CI [−3.855; −0.162]). Results of the subgroup analysis are listed in Table 3 and shown in Figure 4. Adding the variable sex as a covariate into the model does not change any significant results. Conducting the analysis with both sex and baseline fatigue levels as a covariate, the same trend of results can be observed (Supplementary Material 1).

**Table 3:** MANOVA results of the subgroup analysis (impaired cognition vs. intact cognition).

**Descriptive analysis**								
	**Participants with impaired cognition**				**Participants with intact cognition**			
	**HIIT (*****n*** **=** **15)**		**CG (*****n*** **=** **19)**		**HIIT (*****n*** **=** **23)**		**CG (*****n*** **=** **16)**	
	**T0**	**T1**	**T0**	**T1**	**T0**	**T1**	**T0**	**T1**
SDMT (points)	37.8 (8.41)	41.73 (9.69)	32.84 (7.23)	36.16 (8.35)	47.13 (9.25)	50.61 (9.38)	54.25 (10.04)	58.63 (10.86)
VLMT (points)	48.6 (8.67)	51.4 (11.54)	47.37 (10.48)	50.37 (9.73)	54.35 (8.56)	53.61 (10.59)	58.31 (10.51)	55.63 (10.54)
(Total score trials 1–5)								
BVMT-R (points)	14.93 (5.26)	17.27 (5.26)	15.89 (6.91)	15.74 (6.7)	24 (6.37)	21.61 (6.84)	25.06 (6.09)	23.44 (5.57)
**MANOVA**				**ANOVA**				
**Group**	**Cognition**	Group*Cognition			**Group**	**Cognition**	Group*Cognition	
***p-*****Value** ***F-*****Value (df** **=** **3)** **Partial** ***η***^**2**^	***p-*****Value** ***F-*****Value (df** **=** **3)** **Partial** ***η***^**2**^	***p-*****Value** ***F-*****Value (df** **=** **3)** **Partial** ***η***^**2**^			***p-*****Value** ***F-*****Value (df** **=** **3)** **Partial** ***η***^**2**^	***p-*****Value** ***F-*****Value (df** **=** **3) Partial** ***η***^**2**^	***p-*****Value** ***F-*****Value (df** **=** **3)** **Partial** ***η***^**2**^	
0.881	0.013	0.558		SDMT	0.905	0.791	0.512	
0.222	3.857	0.695			0.014	0.071	0.434	
0.010	0.147	0.030			0.000	0.001	0.006	
				VLMT	0.621	0.011	0.544	
				(Total score trials 1–5)	0.247	6.872	0.373	
					0.004	0.091	0.005	
				BVMT-R	0.525	0.025	0.232	
					0.408	5.263	1.457	
					0.006	0.071	0.021	

*Data are presented as mean (standard deviation). HIIT, high-intensity group; CG, control group; T0, baseline; T1, post-intervention; SDMT, Symbol Digit Modalities Test; VLMT, Verbal Learning Memory Test; BVMT-R, Brief Visuospatial Memory Test-Revised*.

## Discussion

This study focused on an analysis of a secondary outcome (cognitive performance) of an original RCT by investigating (i) the effect of HIIT vs. CG on cognitive performance in an overall analysis and (ii) examining the effect of cognitive status (impaired cognition vs. intact cognition) within a subgroup analysis. Results of the overall analysis showed significant time effects for processing speed and verbal learning. Results of the subgroup analysis suggest that effects of exercise training on verbal learning are dependent on cognitive status. In detail, participants classified as cognitive impaired at baseline revealed positive changes in VLMT scores, compared to participants with intact cognition. However, no significant cognition × group interaction was observed. A similar trend was found for visuospatial memory; however, results did not reach statistical significance.

By conducting a subgroup analysis based on the predefined cognitive status of the participants, we considered a common limitation of the majority of exercise studies in this research context. The results strongly support the need of pre-defined inclusion criteria for cognitive performance in exercise intervention studies with pwMS. A major reason why existing studies mostly include participants without assessments of cognitive performance prior to inclusion might be that most of the existing studies do not define cognitive performance as a primary outcome. However, from a methodological point of view, the consideration of cognitive performance at baseline is necessary, as groups with heterogeneous cognitive status achieve varying results, requiring larger sample sizes (24). Baseline memory competence and information processing speed have been shown to be independent predictors of cognitive rehabilitation outcome in MS (25). These findings may partially explain the results of a recent meta-analysis that reported null effects of exercise on global and domain-specific cognitive performance in pwMS. Interestingly, out of 13 included studies only one study (26) evaluated cognitive performance prior to study inclusion. Recruitment of enriched samples of cognitively impaired PwMS are now recommended for cognitive retraining studies in MS (27).

Generally, the evidence for potential effects of exercise training on cognitive performance remains unclear, because the emerging results are inconclusive due to methodological limitations and heterogeneous exercise interventions (14, 16). In a previous published study with similar exercise interventions, significant time × group interactions for verbal learning were identified, indicating that HIIT improved VLMT scores compared to CG. These results are in line with those of Briken et al. (28) who reported significant effects of three different exercise interventions (arm ergometry, rowing, and cycling) compared to a waitlist CG on VLMT scores. The present study did not include a passive or waitlist CG treatment, as these are critical to establish from an ethical point of view in clinical settings like rehabilitation centers. Results of the current study did not confirm those of the previous investigation, which might be explained by the following reasons. The portion of pwMS with impaired cognition relative to the studies' sample size was comparable for the HIIT group. However, the CG group of the current study had a higher portion of pwMS with impaired cognition compared to the previous study indicating, based on the results, that CG also had beneficial impacts on cognitive performance, leading to no interaction effect in the current study. Although only for the SDMT, time effects were observed in both groups accompanied by no group or interaction effect underlying this hypothesis. Moreover, with regard to the subgroup analysis, no group (HIIT vs. CG) effect could be observed, which also indicates no superiority of one exercise regime with regard to cognitive performance. A recently published secondary analysis investigated the effects of a high-intensity aerobic exercise intervention compared to a waitlist control condition on cognitive performance in pwMS, thereby analyzing effects on both the overall sample and a cognitive impaired subsample (29). Results show similar effects to the present analysis, as no interaction effects were observed for the overall analysis. However, based on between-group point estimates, the cognitive impaired subgroup showed clinically significant improvement in SDMT and similar improvements for the selective reminding test.

Besides potential exercise regime independent benefits of exercise on cognition, HIIT applied in this study had a positive impact on physical fitness in pwMS. Against argued worries about potential losses of adherence linked to higher exercise intensities (30), this study showed high adherence rates by pwMS of several disability ranges. However, the setting remains an inpatient rehabilitation that is not comparable to outpatient settings. Concerning the reached exercise intensities, it should be noted that the average HR of the CG was higher than prescribed. This could explain why time × group interaction for rel. VO2peak did not reach significance. Reasons for higher exercise intensities of the CG might emerge since individual exercise intensities were derived from baseline CPET. However, CPET was conducted until the participant's symptom reached maximum so that muscular fatigue, especially in pwMS with higher disability ranges, could occur prior to cardiovascular exhaustion.

This study has some limitations that need to be taken into account when interpreting the results. First, the study was a subgroup analysis, and the division into pwMS with impaired and intact cognition was done afterwards, consequently leaving the original randomization out. However, except for sex (in the subgroup analysis), no baseline differences were observed. Moreover, the sample size calculation was based on the primary outcome of the original trial. However, it is relatively large compared to existing trials in this research context. Second, the study was conducted during inpatient rehabilitation, enhancing adherence toward the exercise interventions but limiting the total time of the intervention period, since a normal stay at the clinic lasts 3 weeks. Consequently, potential neuronal adaptations may not fully develop in that relatively short period of time. Considering the inpatient rehabilitation setting, a passive or waitlist CG was due to ethical reasons not possible to establish. Moreover, it cannot be ruled out that other therapies within the clinical stay may have an impact on the changes in cognitive performance of the participants, especially with impaired cognition, contributing to the observed time effects. Third, test batteries of cognitive performance, such as the BICAMS, might not detect changes of cognitive performance within this short period of time but rather function as an assessment tool to evaluate baseline cognitive function. Fourth, no habituation phase was applied; thus, it cannot be excluded that cognitive performance was biased by learning effects toward habituation of the testing procedures. Fifth, more sensitive methods [e.g., biomarker of neuronal damage, imaging (MRI)] supported by test batteries of cognitive performance potentially reveal more meaningful results. Sixth, since muscular fatigue might bias the results of CPET and derived exercise intensities, other less vulnerable methods should be considered in the future to define exercise intensity. Seventh, although we applied one of the most frequently used and recommended test batteries, we cannot exclude the fact that the results might be linked to ceiling effects. Finally, it should be noted that one intervention group consisted only of female participants since sex was no stratification factor during the randomization process.

Recently published protocols of large-scaled RCTs reveal promising insights into future investigations that consider the limitations of existing studies and function as an example for other upcoming research (31, 32). Moreover, the present subgroup analysis should be enlarged in the future by conducting a prospective RCT, including the same intervention types for both persons with impaired cognition and those with intact cognition.

In conclusion, this study supports the need of RCTs that include cognitive performance as a primary endpoint and define eligibility based on baseline cognitive performance (impaired cognition vs. intact cognition). Future investigations should also conduct a sample size calculation based on the primary outcome of cognitive performance and consider habituation phases and test paradigms that are sensitive enough to detect changes of cognitive performance in a limited period of time.

## Data Availability Statement

The raw data were generated at the Rehabilitation Clinic Valens. Derived data supporting the findings of this study are available from AR upon reasonable request.

## Ethics Statement

The studies involving human participants were reviewed and approved by Ethikkommission Ostschweiz (EKOS): EKOS18/96; Project ID: 2018–01378 Scheibenackerstrasse 4, 9000 St. Gallen. The patients/participants provided their written informed consent to participate in this study.

## Author Contributions

PZ, WB, JK, RG, and JB designed the study. AR, NJ, and SP conducted data acquisition. AR and PZ conducted statistical analysis. AR drafted the manuscript under supervision of PZ and JB. DL gave expert input. All authors revised and approved the manuscript.

## Conflict of Interest

DL discloses research grants/consultancy/speaker bureau from Novartis, Merck, TEVA, Biogen, Sanofi, Bayer. The remaining authors declare that the research was conducted in the absence of any commercial or financial relationships that could be construed as a potential conflict of interest.

## References

[1] Langdon DW Amato MP Boringa J Brochet B Foley F Fredrikson S. Recommendations for a brief international cognitive assessment for multiple sclerosis (BICAMS). Mult Scler. (2012) 18:891–8. 10.1177/1352458511431076 22190573 PMC3546642

[2] Langdon DW Cognition in multiple sclerosis. Curr Opin Neurol. (2011) 24:244–9. 10.1097/WCO.0b013e328346a43b 21519256

[3] Miller E Morel A Redlicka J Miller I Saluk J. Pharmacological and non-pharmacological therapies of cognitive impairment in multiple sclerosis. Curr Neuropharmacol. (2018) 16:475–83. 10.2174/1570159X15666171109132650 29119933 PMC6018194

[4] Motl RW Sandroff BM. Exercise as a countermeasure to declining central nervous system function in multiple sclerosis. Clin Ther. (2018) 40:16–25. 10.1016/j.clinthera.2017.12.001 29287750

[5] Krupp LB Christodoulou C Melville P Scherl WF Pai L-Y Muenz LR. Multicenter randomized clinical trial of donepezil for memory impairment in multiple sclerosis. Neurology. (2011) 76:1500–7. 10.1212/WNL.0b013e318218107a 21519001 PMC3087469

[6] Angevaren M Aufdemkampe G Verhaar HJ Aleman A Vanhees L Physical activity and enhanced fitness to improve cognitive function in older people without known cognitive impairment. Cochrane Database Syst Rev. (2008) 3:Cd005381 10.1002/14651858.CD005381.pub3 18646126

[7] Engeroff T Ingmann T Banzer W. Physical activity throughout the adult life span and domain-specific cognitive function in old age: a systematic review of cross-sectional and longitudinal data. Sports Med. (2018) 48:1405–36. 10.1007/s40279-018-0920-6 29667159

[8] Xu W Wang HF Wan Y Tan CC Yu JT Tan L. Leisure time physical activity and dementia risk: a dose-response meta-analysis of prospective studies. BMJ Open. (2017) 7:e014706. 10.1136/bmjopen-2016-014706 29061599 PMC5665289

[9] Motl RW Pilutti LA Hubbard EA Wetter NC Sosnoff JJ Sutton BP. Cardiorespiratory fitness and its association with thalamic, hippocampal, and basal ganglia volumes in multiple sclerosis. NeuroImage Clinical. (2015) 7:661–6. 10.1016/j.nicl.2015.02.017 25844320 PMC4375633

[10] Kjolhede T Siemonsen S Wenzel D Stellmann J-P Ringgaard S Pedersen BG. Can resistance training impact MRI outcomes in relapsing-remitting multiple sclerosis? Mult Scler. (2018) 24:1356–65. 10.1177/1352458517722645 28752800

[11] Campbell E Coulter EH Paul L. High intensity interval training for people with multiple sclerosis: a systematic review. Mult Scler Relat Disord. (2018) 24:55–63. 10.1016/j.msard.2018.06.005 29936326

[12] Zimmer P Bloch W Schenk A. High-intensity interval exercise improves cognitive performance and reduces matrix metalloproteinases-2 serum levels in persons with multiple sclerosis: a randomized controlled trial. Multiple sclerosis. (2017) 24:1635–44. 10.1177/1352458517728342 28825348

[13] Oken BS Kishiyama S Zajdel D Oberste M Riedel S Kool J. Randomized controlled trial of yoga and exercise in multiple sclerosis. Neurology. (2004) 62:2058–64. 10.1212/01.WNL.0000129534.88602.5C 15184614

[14] Gharakhanlou R Wesselmann L Rademacher A Lampit A Negaresh R Kaviani M. Exercise training and cognitive performance in persons with multiple sclerosis: a systematic review and multilevel meta-analysis of clinical trials. Mult Scler. (2020) 1352458520917935. 10.1177/1352458520917935. [Epub ahead of print]. 32390502

[15] Kalron A Zeilig G. Efficacy of exercise intervention programs on cognition in people suffering from multiple sclerosis, stroke and Parkinson's disease: a systematic review and meta-analysis of current evidence. NeuroRehabilitation. (2015) 37:273–89. 10.3233/NRE-151260 26484519

[16] Sandroff BM Motl RW Scudder MR DeLuca J. Systematic, evidence-based review of exercise, physical activity, and physical fitness effects on cognition in persons with multiple sclerosis. Neuropsychol Rev. (2016) 26:271–94. 10.1007/s11065-016-9324-2 27447980

[17] Joisten N Rademacher A Bloch W Schenk A Oberste M Dalgas U. Influence of different rehabilitative aerobic exercise programs on (anti-)inflammatory immune signalling, cognitive and functional capacity in persons with MS—study protocol of a randomized controlled trial. BMC Neurol. (2019) 19:37. 10.1186/s12883-019-1267-9 30849952 PMC6407211

[18] Polman CH Reingold SC Banwell B Clanet M Cohen JA Filippi M. Diagnostic criteria for multiple sclerosis: 2010 Revisions to the McDonald criteria. Ann Neurol. (2011) 69:292–302. 10.1002/ana.22366 21387374 PMC3084507

[19] Penner IK Raselli C Stocklin M Opwis K Kappos L Calabrese P. The Fatigue Scale for Motor and Cognitive Functions (FSMC): validation of a new instrument to assess multiple sclerosis-related fatigue. Mult Scler. (2009) 15:1509–17. 10.1177/1352458509348519 19995840

[20] Penner I-K Kandziora P Poettgen J Heesen C Lang M Schreiber H German validation of the brief international cognitive assessment for multiple sclerosis (BICAMS) (P5.196). Neurology. (2015) 84(14 Suppl.):P5.196.

[21] Helmstaedter C Lendt M Lux S. Verbaler Lern—und Merkfähigkeitstest [Manual]. Göttingen: Beltz Test BmbH (2011).

[22] Smith A Symbol Digit Modalities Test [Manual]. Torrance, CA: Western Psychological Services (1973).

[23] Benedict RHB Brief Visuospatial Memory Test Revised Professional Manual. Odessa: Psychological Assessment Resources (1997).

[24] Chiaravalloti ND Moore NB Nikelshpur OM DeLuca J. An RCT to treat learning impairment in multiple sclerosis: the MEMREHAB trial. Neurology. (2013) 81:2066–72. 10.1212/01.wnl.0000437295.97946.a8 24212393 PMC3863346

[25] Chiaravalloti ND DeLuca J. The influence of cognitive dysfunction on benefit from learning and memory rehabilitation in MS: a sub-analysis of the MEMREHAB trial. Mult Scler. (2015) 21:1575–82. 10.1177/1352458514567726 25662348

[26] Sebastiao E McAuley E Shigematsu R Adamson BC Bollaert RE Motl RW. Home-based, square-stepping exercise program among older adults with multiple sclerosis: results of a feasibility randomized controlled study. Contemp Clin Trial. (2018) 73:136–44. 10.1016/j.cct.2018.09.008 30243811

[27] DeLuca J Chiaravalloti ND Sandroff BM. Treatment and management of cognitive dysfunction in patients with multiple sclerosis. Nat Rev Neurol. (2020) 16:319–32. 10.1038/s41582-020-0355-1 32372033

[28] Briken S Gold SM Patra S Vettorazzi E Harbs D Tallner A. Effects of exercise on fitness and cognition in progressive MS: a randomized, controlled pilot trial. Mult Scler. (2014) 20:382–90. 10.1177/1352458513507358 24158978

[29] Langeskov-Christensen M Hvid LG Jensen HB Nielsen HH Petersen T Stenager E. Efficacy of high-intensity aerobic exercise on cognitive performance in people with multiple sclerosis: a randomized controlled trial. Mult Scler. (2020):1352458520973619. 10.1177/1352458520973619. [Epub ahead of print]. 33232191

[30] Baquet L Hasselmann H Patra S Stellmann J-P Vettorazzi E Engel AK Short-term interval aerobic exercise training does not improve memory functioning in relapsing-remitting multiple sclerosis-a randomized controlled trial. PeerJ. (2018) 6:e6037 10.7717/peerj.6037 30581662 PMC6295157

[31] Feinstein A Amato MP Brichetto G Chataway J Chiaravalloti N Dalgas U. Study protocol: improving cognition in people with progressive multiple sclerosis: a multi-arm, randomized, blinded, sham-controlled trial of cognitive rehabilitation and aerobic exercise (COGEx). BMC Neurol. (2020) 20:204. 10.1186/s12883-020-01772-7 32443981 PMC7245035

[32] Sandroff BM Diggs MD Bamman MM Cutter GR Baird JF Danielle Jones C. Protocol for a systematically-developed, phase I/II, single-blind randomized controlled trial of treadmill walking exercise training effects on cognition and brain function in persons with multiple sclerosis. Contemp Clin Trials. (2019) 87:105878. 10.1016/j.cct.2019.105878 31704437 PMC6875638

