# Supplementary material for: Cognitive Impairment Impacts Exercise Effects on Cognition in Multiple Sclerosis
Source: Front Neurol. 2021 Jan 28;11:619500. doi: 10.3389/fneur.2020.619500 (PMC7902024; doi:10.3389/fneur.2020.619500)
Supplement: Supplementary file 1 [file Data_Sheet_1.docx]

| **MANOVA** | | | **ANOVA** | | | |
| --- | --- | --- | --- | --- | --- | --- |
| group | cognition | group*cognition |  | group | cognition | group*cognition |
| p-value F-value (df=3) partial η2 | p-value F-value (df=3) partial η2 | p-value F-value (df=3) partial η2 |  | p-value F-value (df=3) partial η2 | p-value F-value (df=3) partial η2 | p-value F-value (df=3) partial η2 |
|  |  |  |  |  |  |  |
| 0.846 0.271 0.012 | 0.023  3.410 0.136 | 0.856 0.257 0.012 | **SDMT** | 0.575 0.318 0.005 | 0.559 0.344 0.005 | 0.722 0.128 0.002 |
|  |  |  | **VLMT  (Total score trial 1-5)** | 0.623 0.243 0.004 | 0.018 5.904 0.081 | 0.996 0.000 0.000 |
|  |  |  | **BVMT-R** | 0.693 0.157 0.002 | 0.045 4.157 0.058 | 0.471 0.525 0.008 |
|  |  |  |  |  |  |  |

Supplement 1: MANOVA results of the subgroup analysis (impaired cognition vs. intact cognition) adjusted for fatigue and sex.

SDMT: Symbol Digit Modalities Test; VLMT: Verbal Learning Memory Test; BVMT-R: Brief Visuospatial Memory Test-Revised
